# Spatio-Temporal Development of Vegetation Carbon Sinks and Sources in the Arid Region of Northwest China

**DOI:** 10.3390/ijerph20043608

**Published:** 2023-02-17

**Authors:** Qifei Zhang, Yaning Chen, Zhi Li, Congjian Sun, Yanyun Xiang, Zhihui Liu

**Affiliations:** 1 School of Geographical Sciences, Shanxi Normal University, Taiyuan 030031, China; 2 State Key Laboratory of Desert and Oasis Ecology, Xinjiang Institute of Ecology and Geography, Chinese Academy of Sciences, Urumqi 830011, China; 3 Research Center of Ecology and Environment in the Middle Reaches of the Yellow River, Shanxi Normal University, Taiyuan 030031, China; 4 School of Public Administration, Shanxi University of Finance and Economics, Taiyuan 030006, China

**Keywords:** vegetation carbon sinks and sources, RSEI, NDVI, FVC, climate change, northwest China

## Abstract

Drylands, which account for 41% of Earth’s land surface and are home to more than two billion people, play an important role in the global carbon balance. This study analyzes the spatio-temporal patterns of vegetation carbon sinks and sources in the arid region of northwest China (NWC), using the net ecosystem production (NEP) through the Carnegie–Ames–Stanford approach (CASA). It quantitatively evaluates regional ecological security over a 20-year period (2000–2020) via a remote sensing ecological index (RSEI) and other ecological indexes, such as the Normalized Difference Vegetation Index (NDVI), fraction of vegetation cover (FVC), net primary productivity (NPP), and land use. The results show that the annual average carbon capacity of vegetation in NWC changed from carbon sources to carbon sinks, and the vegetation NEP increased at a rate of 1.98 gC m^−2^ yr^−1^ from 2000 to 2020. Spatially, the annual NEP in northern Xinjiang (NXJ), southern Xinjiang (SXJ) and Hexi Corridor (HX) increased at even faster rates of 2.11, 2.22, and 1.98 gC m^−2^ yr^−1^, respectively. Obvious geographically heterogeneous distributions and changes occurred in vegetation carbon sinks and carbon sources. Some 65.78% of the vegetation areas in NWC were carbon sources during 2000–2020, which were concentrated in the plains, and SXJ, the majority carbon sink areas are located in the mountains. The vegetation NEP in the plains exhibited a positive trend (1.21 gC m^−2^ yr^−1^) during 2000–2020, but this speed has slowed since 2010. The vegetation NEP in the mountain exhibited only intermittent changes (2.55 gC m^−2^ yr^−1^) during 2000–2020; it exhibited a negative trend during 2000–2010, but this trend has reversed strongly since 2010. The entire ecological security of NWC was enhanced during the study period. Specifically, the RSEI increased from 0.34 to 0.49, the NDVI increased by 0.03 (17.65%), the FVC expanded by 19.56%, and the NPP increased by 27.44%. Recent positive trends in NDVI, FVC and NPP have enhanced the capacity of vegetation carbon sinks, and improved the eco-environment of NWC. The scientific outcomes of this study are of great importance for maintaining ecological stability and sustainable economic development along China’s Silk Road Economic Belt.

## 1. Introduction

The terrestrial ecosystem is a critical component of the global carbon cycle [1]. It is the main platform for atmospheric CO_2_ to enter the terrestrial sphere, and as such is highly vulnerable to climate change and human activities [2,3]. When the vegetation carbon stock is greater than the carbon emitted through soil microbial respiration it behaves as the carbon sink. Whereas the carbon emitted through soil microbial respiration is greater than the vegetation carbon stock and behaves as the carbon source [4]. The net ecosystem productivity (NEP) represents the net carbon exchange between the atmosphere and terrestrial ecosystem, which has been widely used to quantitatively evaluate carbon sinks and sources of terrestrial ecosystems [5,6,7].

Since the start of the 21st century, climate change has greatly impacted the global ecosystem; the increase in CO_2_ concentration significantly promotes rises in temperature and leads to global warming [8]. By 2011, CO_2_ concentrations had increased 40% compared to the mid-19th century, and the average land surface temperature had increased by 0.85 °C [9]. As climate change continues, changes in the eco-environments of arid and semi-arid areas have been drawing increased attention from the international community [2,10,11]. Global drylands account for around 41% of Earth’s land surface [12], and contribute about 40% of global net primary productivity [13]. Semi-arid areas are not only the most sensitive regions to CO_2_ changes [14], but also the largest contributors to the variations in the terrestrial carbon cycle [15]. Their vegetation carbon sinks and carbon sources play a pivotal role in the world’s carbon balance.

Under a warming climate scenario that is exacerbated by human activities, the drylands continue to expand [16,17], intensifying the desertification process [18]. The eco-environment is undergoing drastic changes, such as soil erosion, land salinization, farmland desertification, sharp reduction in green corridors, and ecosystem degeneration [19,20,21]. Human activities have quenched river runoffs, decreased groundwater levels, accelerated desertification, and changed spatiotemporal patterns of land use [21,22]. These changes have a great influence on carbon balance in artificial vegetation, and the disturbed natural vegetation. The expansion of dryland and erosion-induced land degradation may reduce the soil organic carbon storage and increase emission of carbon into the atmosphere [5,23]. The regional drought and heat may reduce plant productivity that need several years to cancel the net uptake of atmospheric CO_2_ [24]. Natural vegetation coverage in arid regions is valuable to climatic change and human activities, while the shrinkage of vegetation causes complex changes in the structure, function, and carbon sinks/sources of the surrounding ecosystems [6,25].

Accurate modeling of the NEP helps to detect the spatial and temporal dynamics of terrestrial ecosystem carbon sinks/sources and their influence factors. It helps to assess the health and the carbon budget and balance of the regional ecosystem. Previous studies have conducted extensive research on the simulation of net primary production (NPP) in Chinese grasslands and its response to climate change. Gross primary productivity (GPP), along with NEP and NPP, are key indicators for characterizing terrestrial ecosystem productivity [26]. Zhang et al. [5] combined the Carnegie-Ames-Stanford Approach (CASA) and empirical models to estimate the NEP in Central Asia and quantitatively evaluate the sensitivity of NEP to climate factors. Currently, the main methods for estimating carbon sinks/sources in terrestrial ecosystems include networked observations, national inventories, model simulations, and atmospheric inversions [27,28,29]. Recent model simulations have been used to compensate for temporal limitations of localized observations in regional and national scales [6,7]. Light energy utilization models, which are based on plant photosynthesis processes and light energy utilization models, have also been used [5,30].

The vegetation ecosystem plays an important role in regional climate change and the global carbon cycle. Forests cover ~30% of Earth’s land surface [31], while grassland covers 40.5% (5.25 billion hectares) and contributes 34% to the total organic carbon of the world’s terrestrial ecosystems (excluding Greenland and Antarctica) [3]. In the arid region, oases have significant cooling effect in summer [32]. Therefore, it is crucial to study the ecological value and contribution of long-term carbon dynamics, as this information is required to accurately assess what needs to be achieved to maintain the global carbon budget balance and provide guidance for greenhouse gas emission mitigation policies.

Globally, northwest China (NWC) is one of the most sensitive areas to climate change in the arid and semi-arid region. It is located in the middle of Eurasia, about 2.5 million km^2^ in extent, with a lower ability to regulate and a highly vulnerable ecosystem. During the past 60-years, the temperature rise has been 0.3 °C/decade in NWC. The number days with a wind speed above 2 m s^−1^ showed a notable negative trend between 1969 and 1992, whereas this trend of wind speed within 1–3 m s^−1^ reversed from 1993 to 2015 [33]. Li et al. [18] detected that the drylands in NWC experienced notable land degradation or vegetation brownness during 1980–2015, and a drying trend has been concluded since the 2000s [25,34], as well as notably enhanced evaporation. Scholars the world over have focused on the region’s climate- and human activity-related challenges, searching for viable solutions. Widely regarded as an important area in the global carbon cycle estimation, the sparse vegetation cover, fragile ecosystems of NWC are extreme sensitivity to global climate change. Chen et al. [25] detected that the vegetation coverage in NWC generally exhibited a positive trend during 1982–1998 and then an abrupt decline since 1998. Recent desert-oasis transition zones in the basin of NWC showed a reduction in area in response to the warming climate and human activities [35]. Deng et al. [36] found that the start time for the phenology growing season was 0.66–3.45 d later than that of previous years under drought condition. In addition to damaging ecosystems, the variations in vegetation carbon sinks and sources can exert growing pressures on economic development and social stability. Presently, numerous studies have been conducted on the characteristics of soil organic carbon mineralization and the differences in soil respiration rate under different land use patterns. However, what are the current dynamic changes of the vegetation carbon cycle and ecological security in NWC in the context of climate change and human activities? What are the changes in the ecosystem carbon cycle and the potential causes?

Vegetation carbon cycle in NWC plays an important role in the global carbon balance. Exploring the dynamic changes of carbon sinks and sources in NWC and evaluating the ecological security is crucial to the security of the arid ecosystem, and ecological civilization construction. Therefore, this study has analyzed the dynamic changes of spatial and temporal patterns of carbon sinks/sources in the terrestrial ecosystems of NWC, based on multi-source remote sensing image data, Normalized Difference Vegetation Index (NDVI), land use data and meteorological data from 2000 to 2020. The NEP calculation model and classifications were used to conduct an ecological security assessment and detect the influencing factors. The main objective of this study was to provide a theoretical basis for maintaining both the ecological stability and economic sustainable development of the Silk Road Economic Belt.

## 2. Materials and Methods

### 2.1. Study Area

**Figure 1:**
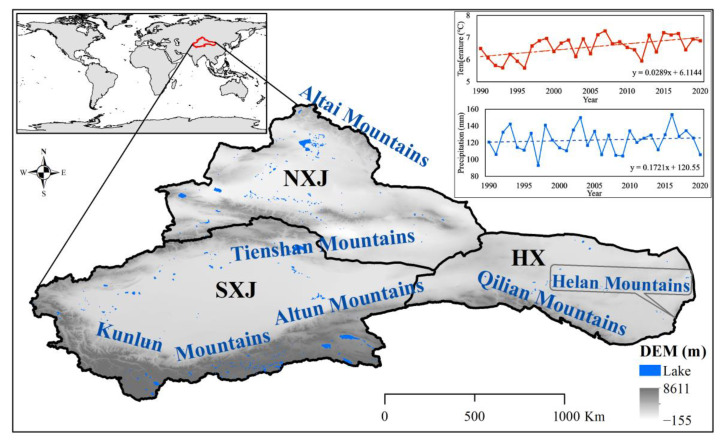
Map of the study area.

Northwest China is located in the Eurasian hinterland and covers a total area of 250 × 10^4^ km^2^. It sits within the boundaries of 73.55°–106.85° E, 34.33°–49.18° N, and includes Xinjiang, parts of Gansu, Inner Mongolia, and Qinghai Province (Figure 1). NWC features several mountain systems, such as the Altai Mountains, Tienshan Mountains, Kunlun Mountains, Karakoram Mountains, Altus Mountains, Qilian Mountains and Helan Mountains. It is characterized by an interphase geomorphic land pattern that includes the Hexi Corridor, Junggar Basin, Tarim River basin, Turpan Basin, Taklimakan Desert, Gobi Desert, as well as other basins and deserts (i.e., the world’s second largest mobile desert–Taklimakan Desert).

Because of its low levels of precipitation, strong evaporation, and vast distance from the nearest ocean, NWC is one of the driest areas in the world. It has sparse vegetation, which is mainly dominated by mountains and desert. The total oasis area accounts for 9.7%, and the desert and Gobi areas accounts for approximately 80% of the region in NWC. A temperate continental climate dominates the region, bringing annual average temperatures of around 6.58 °C and annual average precipitation of 123.30 mm, and the potential evaporation could be up to 2500–3000 mm. The spatial distribution of NWCs water resources is relatively poor and unevenly allotted. Spatially, the water resources are mainly distributed in the western region, and the water resources are less in the eastern region. The resources themselves are mainly composed of glacier and snow meltwater from the mountains. Drought is a common characteristic of this massive arid zone, which has become more and more vulnerable to climate change. The artificial oasis areas in NWC are less than 10% but carry about 98% of the population and produce 95% of GDP. The oasis areas are the main carrier of human activities and economic and social development in NWC.

### 2.2. Materials

Multiple remote sensing data products from the Moderate Resolution Imaging Spectroradiometer (MODIS) data were used in this study for the period 2000–2020. NDVI data were downloaded from the MOD13Q1, with a spatial resolution of 250 m and a temporal resolution of 16 d. The NPP was calculated using the Carnegie-Ames-Stanford Approach (CASA) model, based on the MCD15A3H, MOD15A2H, MCD12Q1, Terraclimate, GLADAS/T3H datasets. The RSEI adopted in the research was estimated using multiple adopted datasets, including MOD09A1 and MOD11A2. Land use data with spatial resolution of 30 m for the periods 2000, 2010, and 2020 were provided by the Resource and Environment Sciences and Data Center, Chinese Academy of Sciences. The land use dataset includes six categories: cultivated land, industrial land, forest, grassland, water, and unused land. The classification standards were interpreted, classified, and digitized according to the land classification system of the Chinese Academy of Sciences, and the decipherment results were verified in conjunction with fieldwork. In this study, the MODIS products and the climate datasets (TerraClimate, GLDAS) during 2000–2020 were downloaded and processed from the Google Earth Engine (GEE) platform. The NPP and RSEI data were calculated by using the GEE platform, the data were processed and resampled at a spatial resolution of 500 m.

The Shuttle Radar Topography Mission (SRTM V4.1) digital elevation model (DEM) with a spatial resolution of 90 m was provided by the Geospatial Data Cloud site, Computer Network Information Center, Chinese Academy of Sciences. Monthly temperature and precipitation data from the Climatic Research Unit (CRU TSV4.06) dataset product (available in 0.5° resolution) was utilized to detect changes in precipitation and temperature since 1990.

All of the datasets used in this study are summarized in Table 1.

### 2.3. Methods

#### 2.3.1. Net Ecosystem Production Algorithm

In this study, the NEP calculation model and classifications were adopted for estimating regional carbon sinks and sources. The scale of vegetation carbon sinks/sources can be expressed as the difference between NPP and soil microbial respiration and carbon emission, without considering other natural environmental and anthropogenic factors. The algorithm to calculate the NEP is:

NEP = NPP − R_H_ (1)

R_H_ = 0.22 × [Exp(0.0913T) + Ln(0.3145P + 1) × 30 × 46.5%] (2)

where NEP is the monthly net ecosystem production (gC m^−2^), NPP is the monthly net primary production (gC m^−2^), R_H_ is the monthly soil microbial respiration (gC m^−2^), T is the monthly mean temperature (°C), and P is the monthly precipitation (mm).

#### 2.3.2. NPP Estimation Using the CASA Model

The vegetation NPP is a key parameter of a terrestrial ecosystem’s carbon cycle and energy flow. It can also be used as an important ecological index to quantitatively evaluate the capability of carbon deposition from vegetation. Although some NPP products can be obtained, the limitations for large regions, complex topography, coarse resolution, and continuous observations occur, e.g., from MODIS products which have missing values across the NWC, with some areas entirely unrepresented, particularly in the lower basins.

In this study, the CASA model proposed by Potter et al. [37] was used to calculate the NPP, which was realized on the GEE platform. This model considers the climate condition (i.e., the NDVI and land use data, temperature, precipitation and solar radiation), and the simulation is determined using two variables: absorbed photosynthetically active radiation (APAR) and light energy conversion (ε). Because of its high accuracy, the CASA model has been widely used to estimate regional vegetation NPP. Compared to other products, the simulated NPP from CASA model showed a close match (R = 0.82, *p* < 0.01) with the NPP from MOD17A3H, which proved better in this study. Detailed information on the model can be obtained from [5,20]. The CASA model is formulated as follows:

NPP (x, t) = APAR(x, t) × ε(x, t)(3)

APAR = SOL × FPAR × 0.5(4)

where NPP (x, t) is the NPP value in pixel x and in month t, APAR is the absorbed photosynthetically active radiation (MJ m^−2^), FPAR is the fraction of photosynthetically active radiation, ε is the light energy conversion (gC M^−1^ J^−1^), and SOL is the total solar surface radiation (MJ m^−2^).

(5) $${\mathrm{FPAR}}_{\mathrm{NDVI}}=\frac{\left(\mathrm{NDVI}\;-{\;\mathrm{NDVI}}_{i,\min}\right)\times \left({\mathrm{FPAR}}_{\max}-{\;\mathrm{FPAR}}_{\min}\right)}{{\mathrm{NDVI}}_{i,\max}-{\;\mathrm{NDVI}}_{i,\min}}+{\mathrm{FPAR}}_{\min}$$

where FPAR_max_ (=0.95) and FPAR_min_ (=0.001) are independent of the vegetation type, NDVI_i,max_ is the NDVI value corresponding to 95% of the NDVI value, and NDVI_i,min_ is the NDVI value corresponding to 5% of the NDVI value. FPAR and SR are calculated as:

(6) $${\mathrm{FPAR}}_{\mathrm{SR}}=\frac{\left(\mathrm{SR}\;-{\;\mathrm{SR}}_{i,\min}\right)\times \left({\mathrm{FPAR}}_{\max}-{\;\mathrm{FPAR}}_{\min}\right)}{{\mathrm{SR}}_{i,\max}-{\;\mathrm{SR}}_{i,\min}}+{\mathrm{FPAR}}_{\min}$$

(7) $$\mathrm{SR}=\frac{1+\;\mathrm{NDVI}}{1-\;\mathrm{NDVI}}$$

where SR_i,max_ and SR_i,min_ are the NDVI_i,max_ and NDVI_i,min_, respectively.

FAPR = αFPAR_NDVI_ + (1 − α) FPAR_SR_ (8)

with α set at 0.5. The light energy conversion ε (gC M^−1^ J^−1^) was calculated as follows:

ε = T_i_ × T_j_ × w_ε_ × ε_max_ (9)

where T_i_ and T_j_ are the low and high temperature stresses on the efficiency of light use, respectively; w_ε_ is the effects of the water stress; and ε_max_ is the maximum light use efficiency (gC M^−1^ J^−1^).

#### 2.3.3. Fraction of Vegetation Cover (FVC) Calculation

The FVC is the percentage of the vertical projection of vegetation (including leaves, stems and branches) on the ground area. It is an important indicator of surface vegetation cover status and has a strong positive correlation with the NDVI. In this study, the FVC in NWC during 2000–2020 was calculated using the mixed-pixel dichotomy model. The assumption of this algorithm is that the NDVI value of each pixel consists of vegetation and soil. The FVC is estimated as follows:

NDVI = FVC × NDVI_Veg_ + (1 − FVC) × NDVI_Soil_ (10)

FVC = (NDVI − NDVI_Soil_)/(NDVI_Veg_ − NDVI_Soil_) (11)

where FVC is the fraction of vegetation cover (%), NDVI_Veg_ is the NDVI value covered by vegetation pixels, and NDVI_Soil_ is the NDVI value of soil or non-vegetation covered pixels. To calculate FVC, we regard the maximum value of the NDVI in the study area as NDVI_Veg_ and the minimum value as NDVI_Soil_. The FVC was divided into five grades: high vegetation coverage area (FVC ≥ 80%), high-medium vegetation coverage area (60 ≤ FVC < 80%), medium vegetation coverage area (40% ≤ FVC < 60%), low vegetation coverage area (20% ≤ FVC < 40%), and bare land area (FVC < 20%).

#### 2.3.4. Remote Sensing Ecological Index (RSEI) Estimation

RSEI is a method to quantitatively evaluate an ecological environment based on remote sensing information and the integration of multiple ecological factors [38,39]. RSEI indicates that the adjustment of four indicators, namely the greenness index (NDVI), wetness index (WET), heat index (land surface temperature, LST) and dryness (normalized difference bare soil index, NDBSI). RSEI can objectively and quickly evaluate the natural ecological environment by using principal component analysis (PCA) [20], which can effectively eliminate the deviation of weight setting by person. The RSEI value ranges from 0 to 1, with a higher value indicating a better eco-environment condition. In this study, the long-term evaluation of eco-environment changes in NWC during 2000–2020 were evaluated by using RSEI. The RSEI algorithm is calculated as follows:

RSEI = f(NDVI, WET, LST, NDBSI)(12)

RSEI_0_ = (1 − {PC1[f(NDVI, WET, LST, NDBSI)]} (13)

RSEI = (RSEI_0_ − RSEI_Min_)/(RSEI_Max_ − RSEI_Min_) (14)

where f represents the combination of the four indicators; NDVI, WET, LST, and NDBSI are the greenness index (present productivity, ecosystem vitality, plant growth), wetness index (reflects the humidity of soil, water and vegetation), dryness index (shows soil drying condition), and heat index, respectively; RSEI_0_ indicates the initial value of the ecological index; PC1 indicates the first component of the principal component analysis; RSEI_Min_ indicates the minimum value of RSEI_0_; and RSEI_Max_ indicates the maximum value of RSEI_0_.

For this study, the RSEI were divided into five grades: worst [0–0.2), poor [0.2–0.4), general [0.4–0.6), good [0.6–0.8), and excellent [0.8–1]. To evaluate the ecological conditions from 2000 to 2020, the RSEI changes in NWC were divided into five grades, as follows: severe degradation (~, −0.2), moderate degradation [−0.2, −0.05), stable [−0.05, 0.05), moderate improvement [0.05, 0.2), and significant improvement [0.2, ~). In addition, the Mann-Kendall trend test [28] was adopted to identify the trends of variables, and the Pearson correlation [29] was used to detect the degree of correlation between the variables.

## 3. Results

### 3.1. Spatial Distribution of Vegetation Carbon Sinks/Sources

**Figure 2:**
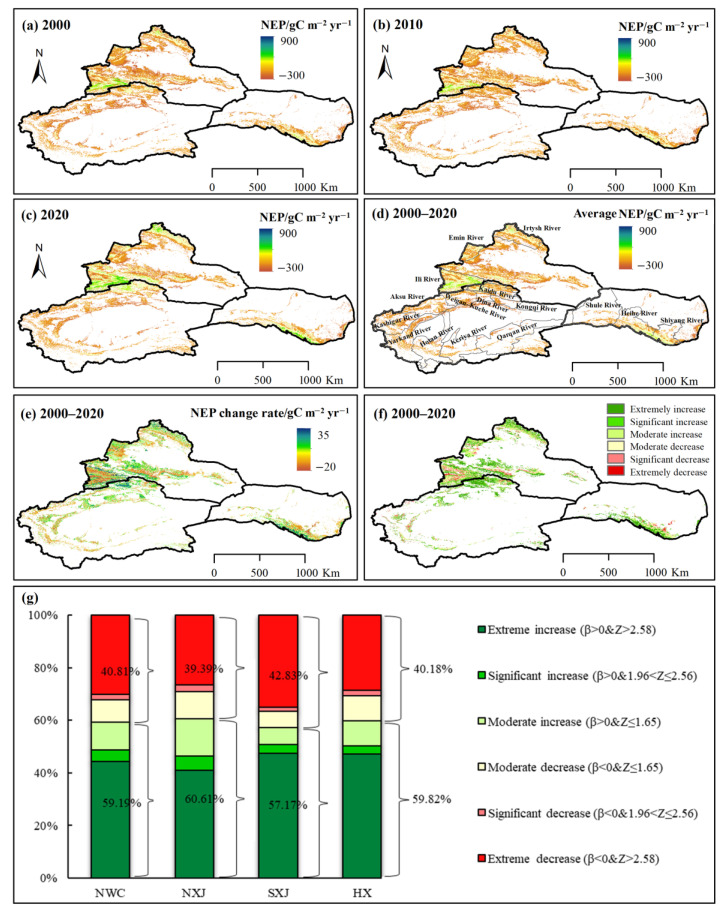
Spatial distribution and variations of NEP in NXJ, SXJ and HX in northwest China. Spatial distribution of NEP during (**a**) 2000, (**b**) 2010, and (**c**) 2020; (**d**) spatial distribution of average NEP during 2000–2020; (**e**,**f**) spatial variations of NEP during 2000–2020; (**g**) Proportion variations of NEP during 2000–2020. (Note: mountain ≥ 1500 m; plain < 1500 m).

The spatial distribution of average carbon sinks and sources in NWC is based on NEP classifications. As shown in Figure 2, the majority of the vegetation-covered region in NWC constitutes carbon sources (NEP < 0) and accounts for 65.78% of the total vegetation area (Figure 2d). Spatially, the NEP values were mainly distributed among mountains, river basins and oases. The high-value areas included the Ili River Valley, the Altai Mountains, the Qilian Mountains, and several river basins, while the low-value areas were mainly distributed in the Taklimakan Desert, Gurbantunggut Desert, Kumtag Desert, and Badain Jaran Desert and its periphery.

The annual average NEP during 2000–2020 ranged from −33.84 to 9.40 gC m^−2^ yr^−1^, giving an annual average of −19.08 gC m^−2^ yr^−1^. The spatial distribution of the annual mean NEP in NWC showed obvious differences (Figure 2). The annual average NEP in northern Xinjiang (NXJ), southern Xinjiang (SXJ), and the Hexi Corridor (HX) was −2.47, −48.36 and −1.92 g C m^−2^ yr^−1^, respectively. Spatially, 58.46% of the vegetation areas in NXJ were carbon sources, 79.91% of the vegetation areas in SXJ were carbon sources, and 57.47% of the vegetation areas in HX were carbon sources (Figure 2d). The vegetation NEP in most basins in SXJ exhibited obvious carbon sources, whereas the Irtysh River, Emin River and Ili River in NXJ were carbon sinks, with an annual average NEP of 3.1, 4.9 and 83.1 gC m^−2^ yr^−1^, respectively.

### 3.2. Temporal Variations of Vegetation Carbon Sinks/Sources

**Figure 3:**
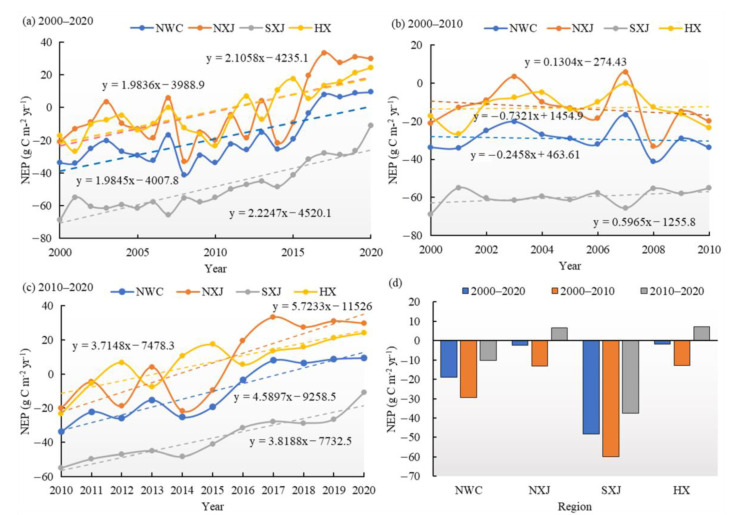
Temporal variations in NEP for different regions of NWC during (**a**) 2000–2020, (**b**) 2000–2010, and (**c**) 2010–2020; temporal variations in annual average NEP during (**d**) 2000–2020, 2000–2010, and 2010–2020.

From 2000 to 2020, the annual average NEP in NWC showed an obvious increasing trend at a rate of 1.98 gC m^−2^ yr^−1^ (*p* < 0.01). As shown in Figure 3, the annual average NEP in NXJ, SXJ and HX increased at rates of 2.11 gC m^−2^ yr^−1^ (*p* < 0.05), 2.22 gC m^−2^ yr^−1^ (*p* < 0.01), and 1.98 gC m^−2^ yr^−1^ (*p* < 0.01). From 2000 to 2010, the vegetation NEP of NWC showed a negative trend of −0.25 gC m^−2^ yr^−1^. Spatially, NXJ’ NEP showed a decreasing trend (−0.73 gC m^−2^ yr^−1^), whereas the vegetation NEP in SXJ and HX showed an increasing trend, with rates rising by 0.60 and 0.13 gC m^−2^ yr^−1^, respectively (Figure 3b). After 2010, the annual average NEP in most regions of NWC showed a significant upward trend of 4.59 gC m^−2^ yr^−1^ (*p* < 0.01). Specifically, the rates for NXJ, SXJ and HX were 5.72, 3.82 and 3.71 gC m^−2^ yr^−1^ (NXJ, *p* < 0.05; SXJ and HX, *p* < 0.01), respectively (Figure 3c).

### 3.3. Spatial Variations of Vegetation Carbon Sinks/Sources

#### 3.3.1. Spatial Variations of Vegetation Carbon Sinks/Sources in Different Basins

As shown in Figure 2e,f, the majority of the vegetation NEP in NWC during 2000–2020 exhibited a positive trend, especially in the mountains. Some 59.19% of the vegetation NEP in NWC showed an increasing trend (Figure 2g), with the highest increases accounting for 4.33% and 44.29% of the total area. Spatially, more than half of the vegetation NEP (60.61%) in NXJ showed an increasing trend, 5.46% and 41.01% of the area showed a significant or extremely significant increasing trend. In SXJ, 57.17% of the vegetation area experienced a rise in NEP, with significant increases and extremely significant increases accounting for 3.31% and 47.38% of the total vegetation area, respectively. Some 59.82% of the vegetation NEP in HX showed an increasing trend, 3.27% and 47.11% of the area showed a significant or extremely significant increasing trend.

From 2000 to 2020, 40.81% of the vegetation NEP in NWC experienced a positive trend. Decreases and extremely significant decreases accounted for 2.02% and 30.14% of the total region area, respectively. Spatially, the decreased vegetation NEP of NXJ accounted for 39.39% of the total area. It was mainly distributed in the junction of the northern Junggar Basin and the Altai Mountains, southern Junggar basin, northern Tienshan Mountains, and the Ili River basin. Some 42.83% of the vegetation NEP in SXJ showed a negative trend, and an extremely decreasing trend was seen in 35.04% of the vegetation NEP. Some 40.18% of the vegetation area in HX showed a negative trend. The vegetation NEP in southeast HX, which includes the Shiyang River basin and the upper reaches of the Heihe River basin, decreased significantly (*p* < 0.01). Throughout the study period, 28.71% of the vegetation NEP showed an extremely significant decrease.

**Figure 4:**
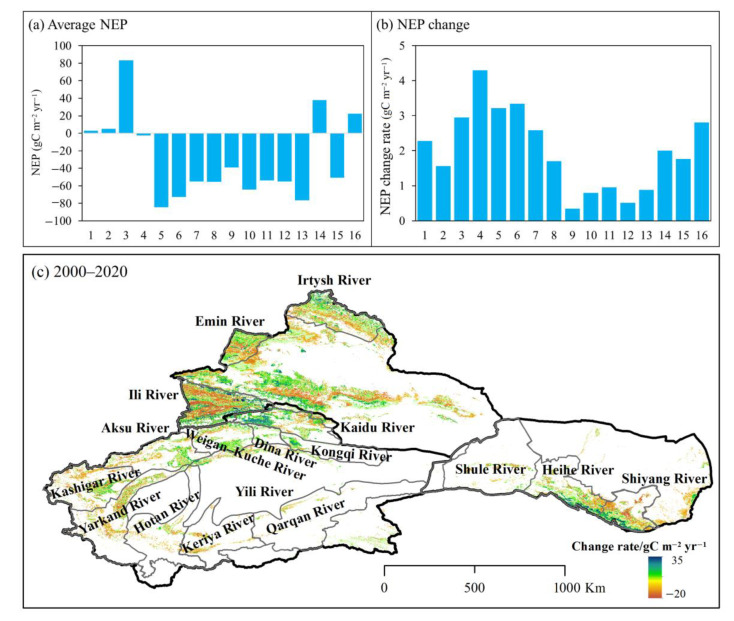
NEP distribution and trends in NWC’ basins during 2000–2020. (**a**) Annual average NEP; (**b**,**c**) spatial variations of NEP in different basins. Note: (1) Irtysh River basin, (2) Emin River basin, (3) Ili River basin, (4) Kaidu River basin, (5) Kongqi River basin, (6) Dina River basin, (7) Weigan-Kuche River basin, (8) Aksu River basin, (9) Keriya River basin, (10) Kashgar River basin, (11) Hotan River basin, (12) Yarkand River basin, (13) Qarqan River basin, (14) Shiyang River basin, (15) Shule River basin, and (16) Heihe River basin.

As shown in Figure 4, the carbon source areas were mainly distributed in the SXJ basins and the western basin of HX during 2000–2020. The annual average NEP in the Kaidu, Kongqi, Dina, Weigan-Kuche, Aksu, Keriya, Kashgar, Hotan, Yarkand, and Qarqan River basins in SXJ were −2.6, −84.5, −72.6, −54.8, −55.4, −38.9, −64.3, −53.8, −55.1, and −76.3 gC m^−2^ yr^−1^, respectively. The annual average NEP in the Shule River basin in HX was −50.7 gC m^−2^ yr^−1^. Conversely, the Irtysh, Emin and Ili River basins in NXJ were carbon sinks, with multi-year NEP averages of 3.1, 4.9, and 83.1 gC m^−2^ yr^−1^, respectively. The annual average NEP values of the Shiyang and Heihe River basins in HX were 37.7 and 22.3 gC m^−2^ yr^−1^, respectively.

Across most of NWC, the NEP showed an upward trend, especially in the basins of the Shiyang, Heihe, and Shule Rivers in HX, the rates of increase were 2.0, 2.8, and 1.8 gC m^−2^ yr^−1^, respectively (*p* < 0.01). Meanwhile, in SXJ, the vegetation NEP increased significantly in the Kaidu, Kongqi, Dina, Weigan-Kuche, Aksu, Kashgar, Hotan, and Qarqan River basins (*p* < 0.01), showing increased rates of 4.3, 3.2, 3.3, 2.6, 1.7, 0.8, 1.0, and 0.9 gC m^−2^ yr^−1^, respectively. In NXJ, the vegetation NEP in the Irtysh, Emin and Ili River basins increased at rates of 2.3 (*p* < 0.05), 1.6, and 2.9 gC m^−2^ yr^−1^, respectively.

#### 3.3.2. Spatial Distributions and Variations of Carbon Sinks/Sources at Different Altitudes

In this study, the arid region of northwest China is divided into mountainous areas (altitude ≥ 1500 m) and plains areas (altitude < 1500 m). As shown in Figure 4, the higher carbon sink areas are distributed across the northern foothills of the Tienshan Mountains and the mid-low altitude areas of the Altai and Qilian Mountains. The annual average NEP of the mountainous areas in NWC was 3.53 gC m^−2^ yr^−1^. The annual average vegetation NEP of these areas in NXJ was the highest (27.48 gC m^−2^ yr^−1^), followed by the HX region (17.01 gC m^−2^ yr^−1^); SXJ was the lowest (−32.71 gC m^−2^ yr^−1^). In plains areas, the highest values of vegetation NEP were mainly distributed in the Ili River Valley and focused in the river basins of SXJ (Figure 4a). The annual average vegetation NEP in NXJ was −30.60 gC m^−2^ yr^−1^, followed by the HX region with an average value of −57.30 gC m^−2^ yr^−1^. The annual average vegetation NEP in SXJ was the lowest, at −76.22 gC m^−2^ yr^−1^.

**Figure 5:**
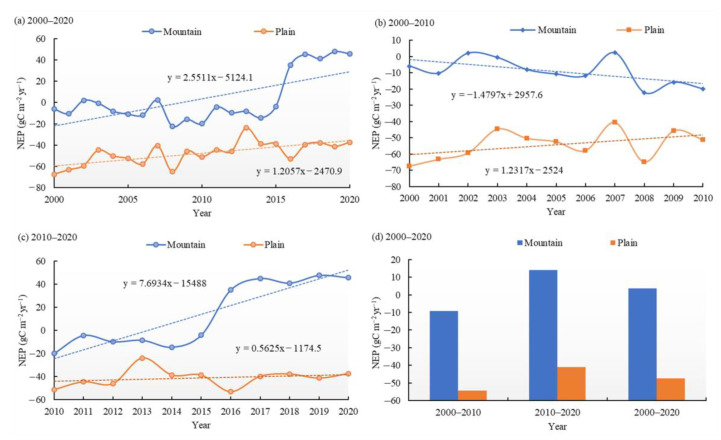
Temporal variations in NEP in the mountains and plains during (**a**) 2000−2020, (**b**) 2000−2010, and (**c**) 2010−2020; (**d**) annual average NEP during 2000−2010, 2010−2020, and 2000−2020.

Throughout the study period, the vegetation NEP values for both the mountains and the plains showed notable positive trends (Figure 5), with increasing rates of 2.55 gC m^−2^ yr^−1^ (*p* < 0.05) and 1.21 gC m^−2^ yr^−1^ (*p* < 0.01), respectively. The vegetation NEP in the plains exhibited a positive trend during 2000–2010, but this speed has slowed since 2010. The vegetation NEP in the mountains exhibited only intermittent changes during 2000–2020; it exhibited a negative trend during 2000–2010, but this trend has strongly reversed since 2010. The annual average vegetation NEP in mountains and plains during 2000−2010 was −9.18 and −54.33 gC m^−2^ yr^−1^, respectively. The NEP in the plains showed an upward trend, at a rate of 1.23 gC m^−2^ yr^−1^, whereas in the mountains it showed a notably decreasing trend, at a rate of 1.48 gC m^−2^ yr^−1^ (*p* < 0.05) during 2000–2010. From 2010 to 2020, the annual average vegetation NEP in mountains and plains again increased, with annual average values of 14.11 gC m^−2^ yr^−1^ and −41.04 gC m^−2^ yr^−1^, respectively. Particularly, the mountainous vegetation NEP showed a significant positive trend of 7.69 gC m^−2^ yr^−1^ (*p* < 0.01), which was higher than that of the plain, with NEP increasing at a rate of 0.56 gC m^−2^ yr^−1^ during 2010−2020. 

**Figure 6:**
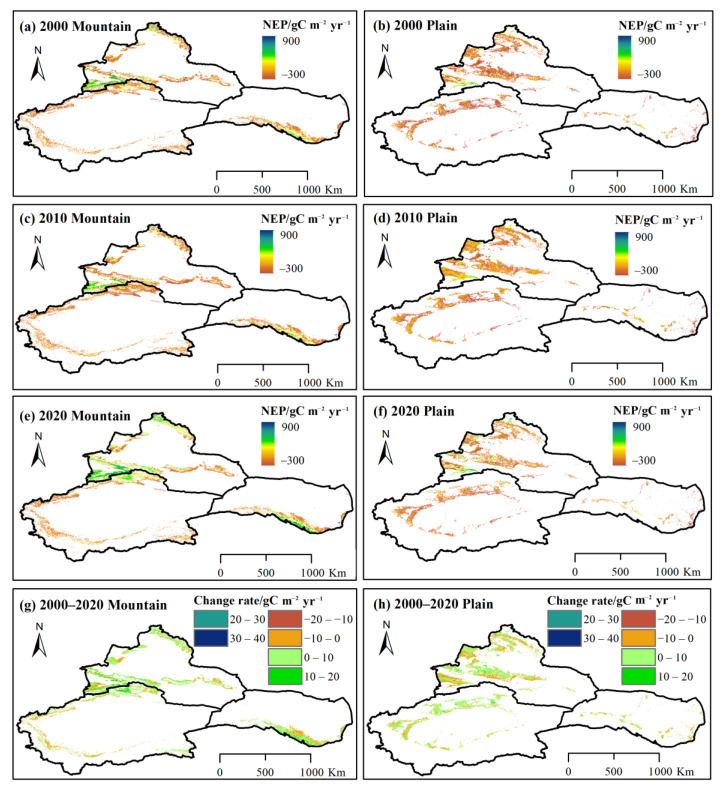
Spatial distribution and variations of vegetation NEP in mountain and plain during 2000−2020. Spatial distribution of NEP in the mountain and plain during (**a**,**b**) 2000, (**c**,**d**) 2010, and (**e**,**f**) 2020; (**g**,**h**) spatial variations of NEP in mountain and plain during 2000−2020.

In terms of the dynamic changes of vegetation NEP in mountain and plain areas during 2000–2020 (Figure 6), the NEP in the NXJ mountain region showed the highest increasing trend, rising at an average rate of 3.17 gC m^−2^ yr^−1^ (*p* < 0.01). In the mountains of SXJ and HX, the NEP increased at rates of 1.88 and 2.52 gC m^−2^ yr^−1^ (*p* < 0.01), respectively. The NEP in the plains also increased substantially in NXJ and SXJ (*p* < 0.01) at a rate of 1.06 and 1.71 gC m^−2^ yr^−1^, respectively. In the plain of HX, the increasing rate of vegetation NEP during 2000–2020 was 0.32 gC m^−2^ yr^−1^. The NEP in the plains of NXJ, SXJ and HX all showed slower trends from 2010 onward (especially since 2015). This is in contrast to the NEP in the mountains, which exhibited high increasing rates of 10.92, 5.16 and 5.13 gC m^−2^ yr^−1^, respectively since 2010.

Obvious geographically heterogeneous distributions and changes occurred in vegetation carbon sinks and sources. Carbon source areas form the majority and are mainly located in plains, which accounts for 54.49% of the total plain area. During 2020, approximately 54.37% of the vegetation NEP in mountains were carbon sinks, whereas 69.81% of the vegetation NEP in the plains were carbon sources. The proportion of carbon sinks of vegetation in mountains increased from 37.43% to 54.37% during 2000–2020, and the proportion carbon sinks of vegetation in the plains ranged from 19.85% to 30.19% during the same period.

## 4. Discussion

### 4.1. Influence Factors of Vegetation Carbon Sinks/Sources Changes

The vegetation carbon sinks and sources changes in the arid and semi-arid regions are driven by numerous factors, including changes in water, temperature, precipitation, evaporation, radiation, vegetation and human activities. The increased precipitation promoted the vegetation growth in Central Asia in the 1990s, but the warming temperatures and decreased precipitation reversed this trend during 1998–2013 [40]. The “warm-wet” pattern was prevailing in HX with a positive trend in NDVI since 1999 [25]. Affected by the climate changes or human disturbances, the regional vegetation carbon sinks in the arid and semi-arid regions will weaken or strengthen; some regions may also be transformed into carbon sinks or carbon sources, e.g., the conversion of natural vegetation to cultivated land could cause rapid decline in soil carbon concentration. The entire desert-oasis ecotone in the Tarim River basin showed a shrinking trend with a large amount of vegetation translated into arable land or poor land since 1990 [35], which has changed the ecological functions and increased the carbon sources. Under the policy of converting farmland to forest or grassland, the sequestration of soil organic carbon has been enhanced, leading to a fall in the soil greenhouse gas emissions in the Loess Plateau [41].

#### 4.1.1. Climate Changes

Climate changes in arid region interact to impose complex and varying limitations on vegetation activities, leading to more carbon emissions from vegetation, soil, cryosphere and water into the atmosphere [42,43,44]. During a comprehensive interpretation of interactive climatic controls on plant productivity in Central Asia, with drought influencing both the carbon budget and balance [6], Chen et al. [45] and Luo et al. [46] concluded that the precipitation in the mountains of Central Asia was increasing at a greater rate than that of the plains, whereas a drier climate was detected in the desert. The characteristics of vegetation NEP in NWC indicated that the coupling effect of precipitation and temperatures were the main drivers of vegetation growth. Yao et al. [22] found that Xinjiang has experienced significant warming and moistening between 1961 and 2018. Zhang et al. [47] detected that the high mountains in Xinjiang were wetter, whereas the deserts had become drier since the 1980s under a warming climate. Spatially, the climate, vegetation as well as the eco-environment in the desert-oasis transition zone showed more sensitivity to a warming climate; as a result, the Taklimakan Desert has expanded 7.8% [48], and the bare land area decreased at a rate of 0.03% yr^−1^ since 1999 [25].

In NWC, the soil heterotrophic respiration (RH) in the vegetation has been positively correlated with temperature (R = 0.36, Table 2). During the study period from 2000 to 2020, temperatures in NWC have an increasing rate of 0.14 °C/decade (*p* < 0.01). Most regions in NWC experienced a warming trend, especially in the far northern portion of NXJ and HX. The rising air temperatures have increased soil respiration and consumed the organic carbon; as a result, it has been consuming the carbon budget of vegetation in NWC. In contrast, the temperatures have shown a rapid rate of increase in Central Asia, which has caused substantial carbon loss [5,6]. Precipitation in NWC showed a rate of 2.28 mm/decade during the study period. The HX region, the south and southeast portions of NXJ, and most areas of SXJ were the main recipients of the increase. Compared to the temperature changes, the vegetation NEP was significantly positively correlated with precipitation, giving correlation coefficients of 0.31. Water is the important limiting factor for ecological stability in arid regions. Hao et al. [49] showed that the vegetation in arid regions is more sensitive to water deficit caused by temperature change due to high water vapor pressure differences and a low soil moisture climate environment. Li et al. [34] found that the vegetation in the Tienshan Mountains of NWC is extremely vulnerable and sensitive to water deficits. Recent studies showed that the temperatures in the mountains are warmer than the lower plains [50,51], which has aggravated the melting of water from glacier and snow [52,53]. Under the influence of climate change, the river runoff in the NWC from the glacier and snow meltwater has greatly increased [52,54,55,56], and this trend will continue. The increasing river runoff replenishment and regulating effect on river runoff will be further enhanced, providing more water resources for the oasis and natural vegetation, thereby helping to improve the ecological environment.

#### 4.1.2. NDVI and FVC Changes

**Figure 7:**
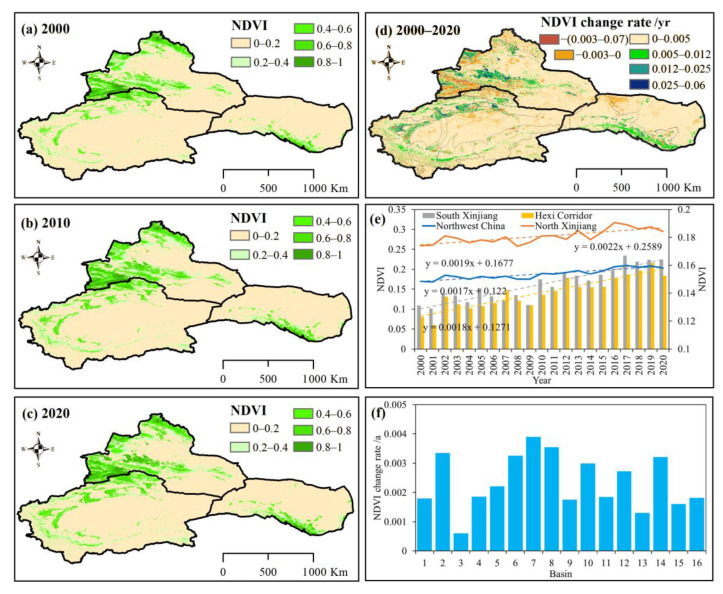
Spatial distribution and variations of NDVI from 2000 to 2020. Spatial distribution of NDVI during (**a**) 2000, (**b**) 2010, and (**c**) 2020; (**d**) spatial variations of NDVI in NWC; (**e**) temporal variations of NDVI in NWC, SXJ, NXJ and HX; (**f**) spatial variations of NDVI across different basins in NWC: (1) Irtysh River basin, (2) Emin River basin, (3) Ili River basin, (4) Kaidu River basin, (5) Kongqi River basin, (6) Dina River basin, (7) Weigan-Kuche River basin, (8) Aksu River basin, (9) Keriya River basin, (10) Kashgar River basin, (11) Hotan River basin, (12) Yarkand River basin, (13) Qarqan River basin, (14) Shiyang River basin, (15) Shule River basin, and (16) Heihe River basin.

Vegetation changes have great influences on the vegetation carbon sinks and sources. The recent increased carbon budget in NWC may result from the improved vegetation. Xu et al. [57] detected that the interactive effects of climate change and land use changes help prediction of the persistence of vegetation change. Liang et al. [58] found that 13.56% of oasis land surfaces in NWC during 1975–2010 showed significant positive trends, whereas 6.07% of oasis land surfaces exhibited a significant negative trend. Wang et al. [59] found that most of the vegetation covered areas in HX experienced significant changes during the period 2001−2017. In this study, the NDVI showed an increasing trend (*p* < 0.01), 88.99% of NDVI exhibited an increase during 2000–2020. The NDVI value rose from 0.17 in 2000 to 0.20 in 2020, representing an increase of 17.65% over 20 years (Figure 7).

Pearson’s correlation coefficient value exhibited a close relation between NDVI and NEP (R = 0.83, *p* < 0.01), and a strong positive correlation with NPP (R = 0.89, *p* < 0.01). The NDVI in NXJ, SXJ and the HX region all showed increasing rates of 0.0022 yr^−1^, 0.0017 yr^−1^, and 0.0018 yr^−1^, respectively (*p* < 0.01). Chen et al. [25] have confirmed these changes, that the vegetation productivity on the northern slopes of the Tienshan Mountains, the southern border of the Tarim River basin, and HX have increased during 2000–2014. Spatially, the basins’ NEP showed a notable increase; NXJ, the Irtysh, Emin, and Ili River basins also showed increasing trends of 0.0018 yr^−1^, 0.0033 yr^−1^, and 0.0006 yr^−1^, respectively (*p* < 0.05). The NDVI in SXJ and HX followed an upward trend as well (*p* < 0.01), rising in the Kaidu, Kongqi, Dina, Weiqian-Kuche, Aksu, Keriya, Kashgar, Hotan, Yarkand, and Qarqan River basins at 0.0019 yr^−1^, 0.0022 yr^−1^, 0.0033 yr^−1^, 0.0039 yr^−1^, 0.0035 yr^−1^, 0.0018 yr^−1^ and 0.003 yr^−1^, 0.0019 yr^−1^, 0.0027 yr^−1^, and 0.0013 yr^−1^, respectively. The increased rates of the NDVI in the Shiyang, Shule, and Heihe River basins in HX were 0.0032 yr^−1^, 0.0016 yr^−1^, and 0.0018 yr^−1^, respectively. The notable increase in FVC provides evidence that the increased NDVI in NWC with positive NEP has promoted vegetation production, as well as enhanced the ability of carbon sinks.

**Figure 8:**
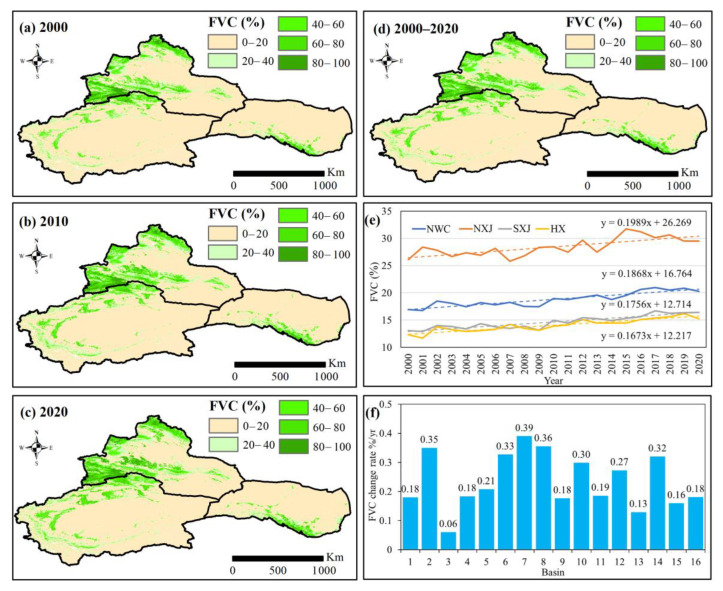
Spatial distribution and variations of FVC from 2000 to 2020. Spatial distribution of FVC during (**a**) 2000, (**b**) 2010, and (**c**) 2020; (**d**) spatial variations of annual average FVC in NWC; (**e**) temporal variations of NDVI in NWC, NXJ, SXJ and HX; (**f**) change rates in NDVI in different basins in NWC: (1) Irtysh River basin, (2) Emin River basin, (3) Ili River basin, (4) Kaidu River basin, (5) Kongqi River basin, (6) Dina River basin, (7) Weigan-Kuche River basin, (8) Aksu River basin, (9) Keriya River basin, (10) Kashgar River basin, (11) Hotan River basin, (12) Yarkand River basin, (13) Qarqan River basin, (14) Shiyang River basin, (15) Shule River basin, and (16) Heihe River basin.

From 2000 to 2020, the FVC in NWC showed a significant increasing trend (0.20% yr^−1^, *p* < 0.01), expanding from 16.96% in 2000 to 20.28% in 2020. Spatially, the FVC in NXJ, SXJ and HX all showed increases, rising at 0.20% yr^−1^, 0.18% yr^−1^, and 0.17% yr^−1^, respectively. In NXJ, the FVC in the Irtysh, Emin, and Ili River basins rose significantly (*p* < 0.05), increasing by 0.18% yr^−1^, 0.35% yr^−1^, and 0.06% yr^−1^, respectively. In SXJ and the HX basins, the FVC likewise exhibited positive trends (*p* < 0.01). Specifically, in the Kaidu, Kongqi, Dina, Weigan-Kuche, Aksu, Keriya, Kashgar, Hotan, Yarkand, and Qanqan River basins, the FVC rose by 0.18% yr^−1^, 0.21% yr^−1^, 0.33% yr^−1^, 0.39% yr^−1^, 0.36% yr^−1^, 0.18% yr^−1^, 0.30% yr^−1^ and 0.19% yr^−1^, 0.27% yr^−1^, and 0.13% yr^−1^, respectively. The expansion rate of the FVC in the Shiyang, Shule, and Heihe River basins was 0.32% yr^−1^, 0.16% yr^−1^, and 0.18% yr^−1^, respectively (Figure 8).

Spatially, as shown in Table 3, the higher and low FVC in NWC showed a notable increase a rate of 0.04% yr^−1^ and 0.05% yr^−1^ (*p* < 0.01), whereas a decreasing trend was detected in the high, medium and lower FVC. The high FVC in HX increased by 0.07% yr^−1^, whereas the high FVC in NXJ and SXJ decreased by 0.002% yr^−1^ and 0.03% yr^−1^, respectively. Moreover, the low FVC in NXJ, SXJ and HX increased at rates of 0.05% yr^−1^, 0.05% yr^−1^ and 0.06% yr^−1^, respectively (*p* < 0.01). A significant positive correlation was detected between FVC and NEP (R = 0.88, *p* < 0.01), and between FVC and NPP (R = 0.93, *p* < 0.01). Both the NDVI and FVC have strong positive trends with NEP, which indicates that the increased FVC have contributed to the capacity of the carbon budget, as well as increasing the carbon sinks.

#### 4.1.3. Human Activities

**Figure 9:**
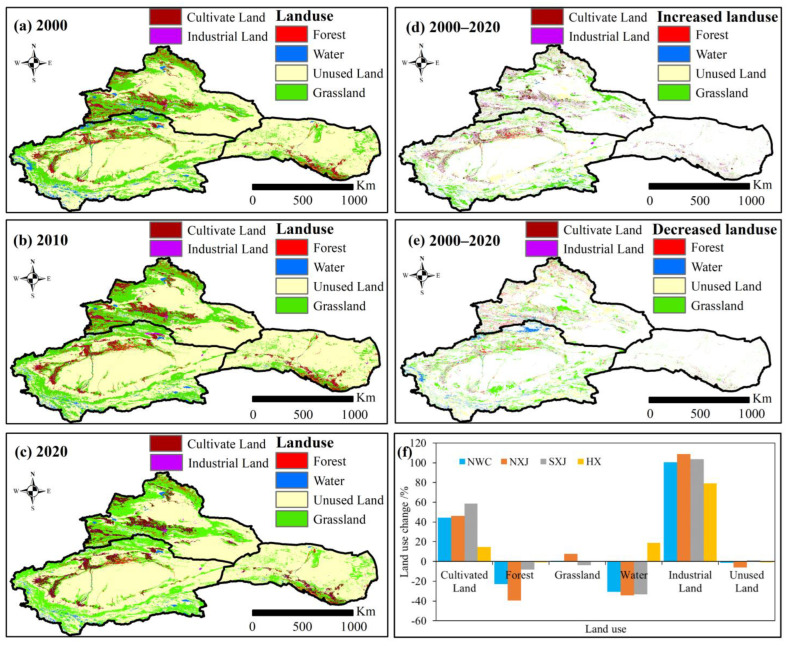
Temporal and spatial variations of land use during 2000−2020. Temporal variations of land use during (**a**) 2000, (**b**) 2010, and (**c**) 2020; (**d**) spatial variations of increased land use during 2000−2020; (**e**) spatial variations of decreased land use during 2000−2020; (**f**) temporal variations of land use during 2000−2020.

Human activities affect the carbon sinks and sources in arid and semi-arid regions by changing the types and areas of land use. The impact of human activities on the vegetation NEP in NWC is primarily through the modification of artificial vegetation NEP. Due in large part to climate change and human activities, the vegetation area (cultivated land, forest and grassland) has expanded by 26,531.27 km^2^ (3.95%), of which cultivated land has expanded significantly (44.50%) from 2000 to 2020 (Figure 9). Spatially, the cultivated land areas in NXJ, SXJ and HX have experienced a particularly notable expansion, increasing by 46.01%, 58.55%, and 14.65%, respectively. The fragile eco-environment of NWC is particularly vulnerable to human disturbance. From 2000 to 2020, the forest area decreased significantly (22.80%), shrinking 39.37%, 8.10%, and 0.87% in NXJ, SXJ, and HX, respectively. Meanwhile, the grassland area in NXJ increased by 7.63%, but grassland in SXJ and HX saw a decrease of 3.62% and 0.28%, respectively. Land use changes are another critical factor affecting soil carbon sequestration or decomposition and greenhouse gas emissions [41]. Under a warming climate and the rapid expansion of the irrigated area, the desert-oasis transition zone showed a reduction in area [25]. Aumuti and Luo [60] found that the total oases areas in Xinjiang increased by 35% from 1990 to 2008. Zhang et al. [55] detected that the oases in the Tarim River basin have greatly expanded during 2000−2020. The deforestation, expansion of cultivated land and urbanization have increased the ecosystem feedback to climate on a large scale. It is necessary to design and control the scale of artificial oases development and protect the space of the natural oases.

### 4.2. Ecological Security Assessment

**Figure 10:**
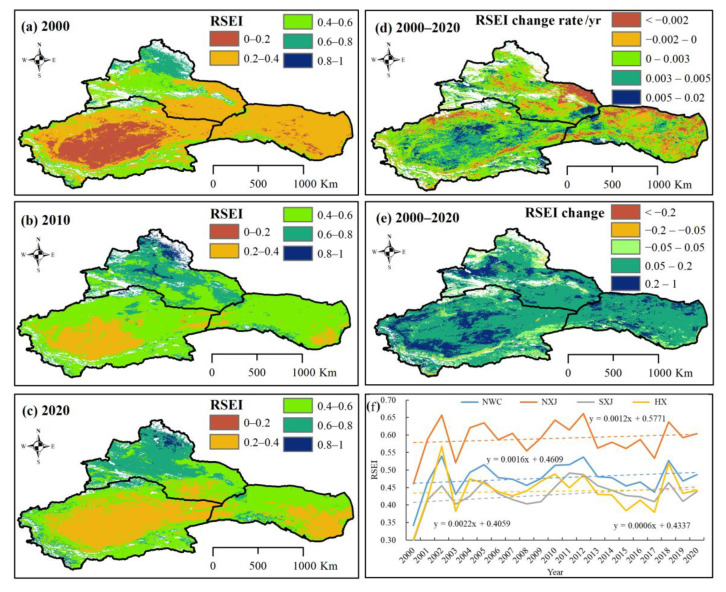
Spatial distribution and variations of RSEI from 2000 to 2020. Spatial distribution in RSEI during (**a**) 2000, (**b**) 2010, and (**c**) 2020; (**d**) spatial variations of RSEI during 2000−2020; (**e**) spatial variations of RSEI between 2000 and 2020; (**f**) temporal variations of RSEI in NWC during 2000−2020.

Under global warming and vegetation changes, the ecological security of NWC has also undergone significant changes. Variations of RSEI in NWC during 2000–2020 are shown in Figure 10. As can be seen, the total eco-environment in NWC greatly improved over the 20-year period, and the ecological grade changed from poor to medium. More specifically, the RSEI increased from 0.34 in 2000 to 0.49 in 2020, and its growth rate increased by 0.0016 yr^−1^. Spatially, the ecological grades in NXJ ranged from medium to good, with the RSEI increasing from 0.46 to 0.60; the RSEIs overall growth rate reached 0.0012 yr^−1^. For SXJ, the eco-environment grade ranged from poor to medium, and the RSEI increased from 0.30 to 0.44, at a growth rate of 0.0022 yr^−1^. The eco-environment grades for HX ranged from poor (RSEI = 0.30) to medium (RSEI = 0.44), and the RSEI in the region increased at a rate of 0.0006 yr^−1^. By using the CA-Markov model, Hou et al. [61] indicated that the ecological environment in the Tarim River basin is expected to steadily improve from 2019 to 2030.

To further detect the driving factors of NEP in NWC during 2000–2020, the relationship between vegetation index (NDVI, FVC, NPP), temperature, precipitation, RSEI, R_H_, and NEP were analyzed in this study (Table 2). A close relation was found between vegetation index (NDVI, FVC, NPP) and NEP in the NWC (R > 0.8, *p* < 0.01), but no obvious correlation was noted in the RSEI, TEM and R_H_, which indicates that the increase in vegetation NEP of NWC has a close positive-exponential relationship to the improved vegetation.

## 5. Conclusions

This study has investigated spatiotemporal changes in the vegetation carbon sinks/sources in the arid region of northwest China and evaluated the ecological security and influence factors of these changes. Our preliminary conclusions are as follows:

The distribution of vegetation carbon sinks and sources showed obvious spatial and vertical zonal characteristics. Approximately 65.78% of the vegetation area in NWC were carbon sources. Spatially, 58.46% in NXJ were carbon sources, and 79.91% in SXJ were carbon sources. Of the vegetation area in HX, 57.47% were carbon sources. The carbon sources are mainly distributed in plain areas with an annual average NEP of −47.52 gC m^−2^ yr^−1^, while the carbon sinks are primarily located in mountainous areas with an annual average NEP of 3.53 gC m^−2^ yr^−1^.

The annual average carbon capacity of vegetation in NWC changed from carbon sources to carbon sinks from 2000 to 2020. The annual average vegetation NEP ranged from −33.84 in 2000 to 9.40 gC m^−2^ yr^−1^ in 2020, with an increased rate of 1.98 gC m^−2^ yr^−1^. This trend has been more notable since 2010, at a rate of 4.59 gC m^−2^ yr^−1^. Spatially, the annual NEP in NXJ, SXJ and HX rose 2.11, 2.22 and 1.98 gC m^−2^ yr^−1^, respectively during 2000−2020. The vegetation NEP in the plains showed positive increases of 1.21 gC m^−2^ yr^−1^ from 2000 to 2020. However, the vegetation NEP in the mountain regions showed a notable negative trend of –1.48 gC m^−2^ yr^−1^ from 2000 to 2010; the downward trend was reversed to a notable upward trend at rate of 7.69 gC m^−2^ yr^−1^ after 2010.

The capability of vegetation carbon deposition in NWC was enhanced, with the declined carbon sources and enhanced carbon sinks from 2000 to 2020. The spatial distribution of the carbon sinks/sources change matches the improvement in the eco-environment. The entire eco-environment of NWC received a boost, as was evident from the rise in ecological grades from poor to medium. The RSEI increased from 0.34 in 2000 to 0.49 in 2020, resulting in a 72.97% improvement in its eco-environment. There were notable increases in the NDVI (increased from 0.17 to 0.20), the FVC rose from 16.96% to 20.28%, and the NPP jumped by 1.59 gC m^−2^ yr^−1^. Spatially, a better eco-environment has been emerging, especially in the mountainous basins. In the context of climate change and human disturbances, the eco-environment of NWC has an overall positive outlook, with some drawbacks. The spatial distribution of the carbon sinks/sources change matches the spatial distribution of vegetation changes. The NDVI, FVC and NPP also showed improvements, which have contributed to the increase in vegetation carbon sinks and the improvement of the eco-environment. Recent improvements in the ecological service function, and the increased ecosystem service value under the climate conditions have a great influence on carbon sinks/sources in NWC.

The variations in vegetation carbon sinks and sources in NWC are controlled by climate change, human activity, topography characteristic, policy, etc. Due to the increasing water resources, natural vegetation and land-type there has been considerable changes. Population increase has stressed the utilization of water resource and promoted the transformation of natural vegetation coverage to artificial and industrial land. There is an urgent need to strengthen the coordinated management of water resources and protection of ecological security in NWC. It is important to protect and restore the ecosystem and maintain the health of the ecological service function. Determine and control the scale of oases to a reasonable spatial area. The recovery of the natural vegetation is needed to enhance the capacity of soil organic carbon and reduce the emissions of soil greenhouse gas. Water resources are the limiting factor for ecological attributes in arid and semi-arid areas in NWC, and they are crucial to support the economic and social development and maintain the stability of the eco-environment. There is a need to strengthen the management and optimize the utilization structure of water resources, and improve the utilization efficiency in the arid region. Optimize water rights management, strengthen oasis land resources and control the development of groundwater. In the process of restoration and improvement of the damaged ecosystem, it is necessary to accelerate ecological restoration and improve ecological functions. Further research is needed to provide a theoretical basis and scientific support for building a life community of mountains, plains, rivers, lakes, forests, cultivated land, grass, sand and ice in harmony between humans and nature.

## Figures and Tables

**Table 1:** Data product types and sources.

Data Products	Variables	Spatial Resolution	Temporal Resolution	Data Sources
MOD13Q1	NDVI	250 m	16 d	https://modis.gsfc.nasa.gov/ accessed on 27 September 2022
MOD09A1	SR	500 m	8 d	https://modis.gsfc.nasa.gov/ accessed on 12 November 2022
MOD11A2	LST	1 km	8 d	https://modis.gsfc.nasa.gov/ accessed on 12 November 2022
MOD15A3H	FPAR	500 m	4 d	https://modis.gsfc.nasa.gov/ accessed on 27 September 2022
MOD17A3H	NPP	500 m	Yearly	https://modis.gsfc.nasa.gov/ accessed on 10 January 2023
MCD12Q1	Landcover (IGBP)	500 m	96 d	https://modis.gsfc.nasa.gov/ accessed on 27 September 2022
TerraClimate	Pre/SOL	4 km	Monthly	https://www.ecmwf.int accessed on 27 September 2022
T3H(GLDAS)	Tem	0.25°	3 h	http:/ldas.gsfc.nasa.gov/ accessed on 27 September 2022
LUCC Data	Landcover	30 m	5 year	http://www.resdc.cn/ accessed on 25 August 2022
CRU TSV4.06	Pre/Tem	0.5°	Monthly	https://crudata.uea.ac.uk/cru/data/hrg/#info accessed on 19 September 2022
SRTM	DEM	90 m	-	https://glovis.usgs.gov/ accessed on 2 September 2022

Note: SR (surface reflectance); LST (land surface temperature); FPAR (fraction of photosynthetically active radiation); Pre (precipitation); Tem (temperature); SOL (total solar radiation).

**Table 2:** Annual average correlation coefficients between NEP, NPP, NDVI, FVC, RSEI, TEM, PRE and R_H_ in NWC from 2000–2020.

Variables	NEP	NPP	NDVI	FVC	RSEI	TEM	PRE	R_H_
NEP	1	0.96 **	0.83 **	0.88 **	0.08	0.30	0.31	0.18
NPP		1	0.89 **	0.93 **	0.10	0.29	0.40	0.32
NDVI			1	0.98 **	0.19	0.23	0.42	0.43
FVC				1	0.25	0.24	0.44 *	0.38
RSEI					1	−0.14	0.13	0.10
TEM						1	−0.06	0.36
PRE							1	0.48 *
R_H_								1

Symbols: * Significance *p* < 0.05; ** Significance *p* < 0.01.

**Table 3:** FVC changes in different grades in NWC during 2000–2020.

Region	Variables	High FVC	Higher FVC	Medium FVC	Lower FVC	Low FVC
NWC	Average (%)	84.79	69.77	49.86	28.11	9.04
	Slope	−0.01	0.04	−0.005	−0.01	0.05
	Z value	0	4.62 **	−1.96 *	−2.81 *	4.92 **
NXJ	Average (%)	84.93	70.37	49.86	28.24	10.73
	Slope	−0.002	0.04	−0.004	−0.02	0.05
	Z value	0.27	4.14 **	−0.75	−1.90	3.96 **
SXJ	Average (%)	84.19	68.53	49.86	28.17	8.11
	Slope	−0.03	0.08	−0.002	−0.004	0.05
	Z value	−2.14 *	3.71 **	−0.63	−1.60	4.74 **
HX	Average (%)	83.41	69.76	49.90	27.51	9.28
	Slope	0.07	0.06	−0.02	−0.01	0.06
	Z value	3.96 **	3.59 **	−2.39 *	−0.75	4.38 **

Symbols: * Significance *p* < 0.05; ** Significance *p* < 0.01.

## Data Availability

Not applicable.
